# Primarily Proximal Jejunal Stone Causing Enterolith Ileus in a Patient without Evidence of Cholecystoenteric Fistula or Jejunal Diverticulosis

**DOI:** 10.1155/2016/8390724

**Published:** 2016-10-10

**Authors:** Houssam Khodor Abtar, Mostapha Mneimneh, Mazen M. Hammoud, Ahmed Zaaroura, Yasmina S. Papas

**Affiliations:** ^1^Makassed General Hospital, Department of Surgery, Beirut, Lebanon; ^2^Saint George Hospital University Medical Center, Department of Surgery, Beirut, Lebanon

## Abstract

Stone formation within the intestinal lumen is called enterolith. This stone can encroach into the lumen causing obstruction and surgical emergency. Jejunal obstruction by an enterolith is a very rare entity and often missed preoperatively. To our knowledge, most cases of jejunal obstruction, secondary to stone, were associated with biliary disease (cholecystoenteric fistula), bezoar, jejunal diverticulosis, or foreign body. Hereby we present a rare case report of small bowel obstruction in an elderly man who was diagnosed lately to have primary proximal jejunal obstruction by an enterolith without evidence of a cholecystoenteric fistula or jejunal diverticulosis. This patient underwent laparotomy, enterotomy with stone extraction, and subsequent primary repair of the bowel.

## 1. Introduction

The term enterolithiasis defines intestinal intraluminal stone. This pathology can also be described as enterolith ileus or pseudogallstone ileus when it causes small bowel obstruction [1]. Extrinsic, intramural, and intraluminal causes are all possible etiologic factors of small bowel obstruction where postoperative adhesions remain the most common cause and account for 74% of all cases [2]. Gallstone ileus accounts for only 1–4% of cases [3]. Till year 2011 only 39 cases of primary jejunal enterolithiasis resulting in small bowel complication had been reported and the majority of them are related to jejunal diverticulosis [4].

## 2. Case Report

A 76-year-old male patient presented to our emergency department with a 72-hour history of persistent nausea, vomiting, and generalized fatigue associated with diffuse colicky abdominal pain. He had a long history of intermittent episodes of abdominal pain and distension. He was afebrile and obstipated and did not pass stool for 3 days. His past medical history is significant for hypertension and prostate cancer. He had open prostatectomy 1 year ago. Upon physical exam, the patient was hemodynamically stable and slightly dehydrated. His abdomen was soft with mild diffuse tenderness and distention.

Blood tests revealed a leukocyte count of 11,500 × 10^9^/L (neutrophils 92%), C-reactive protein of 36 mg/L, and Cr of 2 mg/dL. The remaining lab studies were within normal limits. We did not order a plain abdominal film as this would not show valuable information regarding the diagnosis.

**Figure 1:**
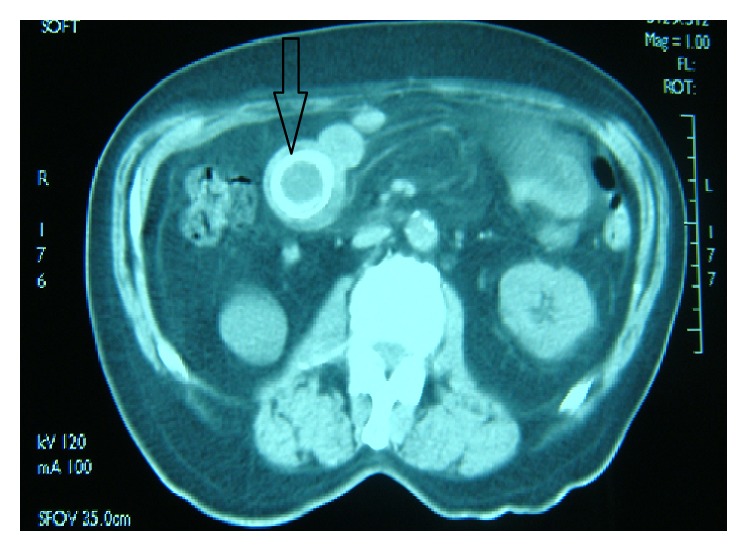
Contrast-enhanced computed tomographic image showing dilated jejunal loop (up to 5 cm in diameter) with a large calcified ring (black arrow).

A computed tomography scan with oral contrast was performed and showed proximal dilated jejunal loop (up to 5 cm in diameter) with a large ring of calcification likely suggestive of ascariasis (Figure 1); the rest of the bowels had a normal caliber. The gall bladder was not distended and there was neither air in the biliary tree (pneumobilia) nor free fluid within the abdominal cavity.

The patient was admitted to the hospital. Conservative management was started with IV hydration, pain management, and a nasogastric tube (drainage of 1.5 L bilious material). He was prepared for exploratory laparotomy through a supraumbilical midline incision.

**Figure 2:**
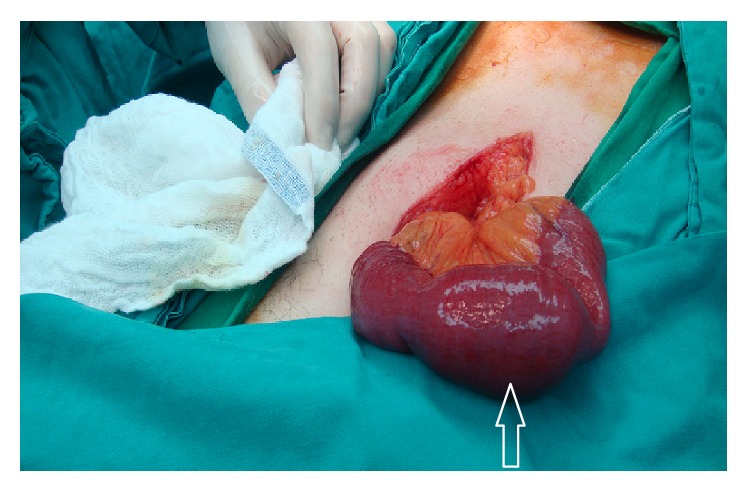
Intraoperative findings. Impacted proximal jejunal stone causing obstruction (white arrow).

**Figure 3:**
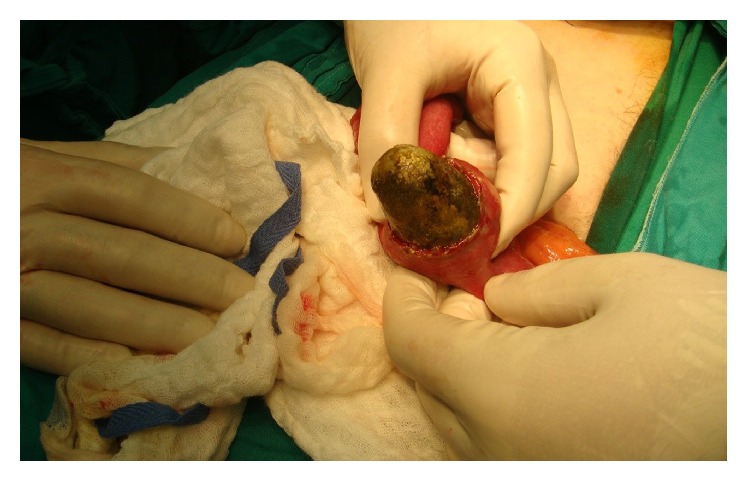
Large stone extracted from within the jejunal lumen by enterotomy with subsequent primary closure.

Upon surgical exploration, the proximal jejunum was found to be dilated, whereas the distal jejunum, ileum, and large bowels were collapsed. A mass about 5.5 cm in size was found to be obstructing the proximal jejunum about 30 cm from the ligament of Treitz (Figure 2). An enterotomy was performed directly over the mass and a large stone was extracted from within the jejunal loop (Figure 3). The opening was closed primarily. The gall bladder appeared normal without evidence of cholecystoenteric fistula. And even so we did not find any jejunal diverticula after complete running of the jejunum. Patient had a smooth postoperative course discharged home without any consequences.

## 3. Discussion

Proximal jejunal obstructions are typically caused by adhesions or tumors. Less frequently, such cases can be secondary to strictures because of inflammatory bowel disease, gallstone impactions, bezoars, and/or foreign bodies [5]. To our knowledge, most cases of stone-related small bowel obstruction described in the literature were secondary to cholecystoenteric fistulae most commonly located at the level of the terminal ileum [6]. Obstruction at the level of the jejunum by a stone in the absence of a cholecystoenteric fistula, like in the case reported here, has been very rarely reported.

Some authors have found a possible association between primary enteroliths in the jejunum and the presence of small bowel diverticuli. With the usual composition of primary enterolith being choleic acid, an end product of bile salt metabolism, it has been postulated that the formation of these stones is secondary to the acidic pH shift within the small confined bowel diverticulum [7].

Another contributing factor that has been reported is the stasis encountered in patients with bowel hypomotility. Indeed, stasis seems to be necessary to permit the progressive accumulation of particulate matter leading eventually to the formation of a stone [3]. In our case this mechanism played an important role in stone formation.

Other possible causes of enterolith in the small bowel include Meckel's diverticulum, small bowel anastomosis, metabolic diseases, intussusception, intestinal strictures, and inflammatory or infectious enteritis [7]. Congenital defects, such as luminal atresia, stenosis, or intestinal aganglionosis, are of the most common causes of small bowel stone formation in the pediatric population [8].

Concerning the management of such cases crushing the enterolith and milking it distally is the first step to do [7]. If this fails, enterotomy is considered then by most experts to be the standard procedure for the management of mechanical small bowel obstruction by a stone, because conservative management has been found to be frequently unsuccessful [9].

In conclusion, jejunal obstruction by a primary enterolith is a very rare entity. This pathology should be expected when other common pathologies have been excluded for the cause of small bowel obstruction in the elderly population. Hence, diagnosis and management are often delayed. Surgical exploration is often necessary as it can result in serious potential complications.
